# Ambient scribes in general practice — help or hindrance?

**DOI:** 10.3399/BJGP.2026.0097

**Published:** 2026-08-01

**Authors:** Emma Ladds, Eleanor Barry, Sharon Dixon, Francesca H Dakin

**Affiliations:** 1 Nuffield Department of Primary Care Health Sciences, University of Oxford, Oxford, UK

## Introduction

The recent *Fit for the Future: 10 Year Health Plan for England* promises a techno-utopian future. The NHS will be liberated from efficiency challenges, resource constraints, and the supply-and-demand gap by new, integrated technological advancements.^1^ Similar convictions have inspired governments since the introduction of the computer in the 1980s, resulting in electronic health records (EHRs), algorithmic guidelines, systematised data collection, and centralised incentivisation systems.

Such advances have transformed primary care. EHRs and population-level data have strengthened epidemiological understanding, supported clinical trials, and informed more targeted treatment and prevention strategies. Digital access and remote consulting have expanded routes into care and increased the speed and efficiency of communication. Technological platforms have enabled greater workload distribution, efficacy of administrative and transactional tasks, and contributed to greater activity.^2^

However, there have been unintended consequences. Care has become increasingly transactional and fragmented,^3^ compromising both quality of care *and* staff wellbeing.^4,5^ Increasing activity has not necessarily translated into improvements in outcomes, and inequalities have been exacerbated by variable digital literacy and infrastructure.^5^ Moreover, quantifiable, technological knowledge has been promoted above human instincts, experience, and values, leading to what some have called a *‘crisis of care’* within the NHS.^6^

Artificial intelligence (AI) tools have the potential to intensify both the benefits *and* challenges of technology. One recent study of general practice patients and staff revealed a belief that AI could reduce clinician workload, speed up patient access, and improve safety by supporting triage, prioritisation, and decision making.^7^ However, participants also highlighted the risk of depersonalising care, raised concerns about safety in complex cases and the reliability of patient data entry, and emphasised the need for strong clinical oversight to maintain trust and prevent errors.

Here we consider some of the major arguments made for ‘AI scribes’ in general practice. These use ambient intelligence to transcribe and summarise consultations, generating structured notes and inputting information into EHRs. It is argued they will improve efficiency, increase the accuracy of notes, support patient participation in their health records, and yield system-wide benefits. While acknowledging this potential — and the evolutionary nature of such technologies, which will inevitably result in future improvements — we argue that the challenges, risks, and losses arising from their introduction also need careful consideration.

## The ‘efficiency’ argument

General practice remains the bedrock of the UK NHS. However, it is under increasing strain as the number of full-time equivalent GPs continues to decline,^8^ while demand increases.^9^ Sequential governments facing this challenge have increased the number of non-GP professionals within general practice, expanded the range of other community providers, and encouraged the use of digital triage and signposting approaches. These changes have challenged Starfield’s definition of high-quality general practice as that which provides first-contact care, offering continuity, coordination, and comprehensiveness,^10^ as holistic caring has been fragmented into a series of transactional, distributed tasks.^11^

A relational approach to consultations, particularly within the context of longitudinal continuity, offers numerous efficiencies.^12,13^ Patients describe such consultations as those where the doctor made time to listen or empathise, legitimising their need and allowing them to fully engage. Encounters are more satisfying and therapeutically effective.^14^ Longer GP appointments have been associated with better recognition and management of psychosocial issues and patient enablement;^15,16^ however, given the ageing, multimorbid nature of the UK population and range of complex medical options and decisions, time in UK GP consultations is highly pressured.^17^

AI scribes are marketed as tools to produce faster, accurate documentation, freeing up time for greater relational connection. While this could be true in administrative or transactional encounters, for example, consultations for phlebotomy, minor procedures, or more protocolised tasks, such technologies also risk introducing false efficiencies. Not only are AI transcripts longer and *more* time consuming to read, but GPs consistently report needing to re-read, restructure, and correct AI-generated notes.^18^ Their use increases after-hours work, suggesting cognitive and workflow disruption before any efficiency gains.^19^ Indeed, GPs report using any time saved to prevent burnout rather than expanding activity or relational connectivity.^19^ Moreover, much relational communication is non-verbal and subtle. Non-lexical sounds such as ‘mm-hm’ or ‘uh-uh’ are poorly captured by AI scribes, resulting in distorted, inaccurate, and superficial records,^20^ and raising concerns about safety and clinical reasoning.^21^ Many tools do not support diverse languages,^18^ with performance varying by accent, dialect, and patient characteristics, thus compounding digital exclusion and inequalities.

These functionality issues are likely to improve over time, but there remains a risk that AI-generated transcripts will undermine the fundamental role of the patient record. Note-taking is not just an account; it is a form of interprofessional clinical analysis that processes embodied, relational, and longitudinal knowledge. An AI-generated summary will inhibit this, producing superficial digital facsimiles of encounters and individuals,^5^ rather than a holistic representation of a longitudinal process of care.^22^ Such isolated snapshots, lacking context or nuance, may be incomplete or misleading, thus misshaping professional judgements.^5^

Additionally, clinicians may lose the cognitive benefits afforded by the reflective space that note-taking provides. The re-ordering of events, articulation of uncertainty, noticing what has not been said, reconsidering diagnoses or investigations, and reviewing colleagues’ entries all contribute to clinical reasoning, team sense-making, and tacit calibration of risk. Such losses risk offsetting any time saved and introducing the potential for automation bias to encourage uncritical acceptance of AI-generated notes.^22,23^

The circumstances in which the tools are developed exacerbate these challenges. Tools are imbued with biases that come to structure how they can (and cannot) function.^5^ Khera and colleagues, for example, have described how the content within AI-generated records, and the nature of the consultations they support, reflect the tool’s design.^23^ This is shaped and optimised by the underpinning business models, which conceptualise efficiency in terms of speed and throughput. AI tools in healthcare settings risk privileging interactions that are readily structured, standardised, and measurable. Clinicians whose workflows already align with transactional or protocol-driven decision making may be particularly likely to rely on them.^24^ This generates feedback loops where the tool’s evolution is informed by ‘transactional data’, thus reinforcing these patterns of care.^24,25^ This is helpful in the contexts of the aforementioned administrative or procedural encounters, but there may be an ideological mismatch in interactions that include more interpersonal, relational components.

## The ‘accuracy’ argument

AI transcription is also promoted to improve the accuracy and utility of the EHR through automated classification and coding. While this may improve the interrogatability of EHRs, offering administrative, clinical, and research benefits, it also poses challenges. Many diagnoses evolve in general practice over sequential consultations. The initial presentation, often shrouded in uncertainty, frequently presents multiple potential classifiable outcomes that are refined over time and through investigation. Automated coding formalises uncertain or narrative elements into rigid categories,^18^ risking misclassification and incorrect diagnoses, which affect clinical care, quality metrics, and population health data.^22^

The drive for classification also overlooks the complexity of symptoms in primary care, conflating them with diagnoses. As early as 1974, Thomas described 40% of patients presenting to general practice to whom a formal diagnostic label could not be attached.^25^ Not only are such patients at risk of overinvestigation, but the approach of the GP is crucial in determining their outcome.^26^ A holistic approach that bears witness to suffering beyond a reductive biomedical approach can offer significant therapeutic benefit.^27^ AI tools risk undermining this, potentially harming individuals and the wider system.

Moreover, online triage platforms and AI-driven templates standardise input *in order* to produce codable outcomes. While a necessary feature of the tool, this ‘user configuration’ constrains patients to categories, limiting expression and interpretation.^5^ It also triggers a feed-forward cycle, whereby practice becomes increasingly focused on biomedical consultation features as this is what the tools have been ‘trained’ to promote. Nuanced elements (non-verbal cues, ambivalence, layered narratives, or safeguarding subtleties) are disadvantaged because they resist structured capture. If AI scribes are switched off or perform less well for such complex encounters, they are less prominent in the training data. The result is a model that performs best for straightforward, codifiable presentations and least well where clinical risk and vulnerability are greatest — a fundamental remit of general practice.

## The ‘ownership’ argument

In the UK, NHS Digital is the national custodian for general practice health records. The GP practice acts as the initial data controller, responsible for handling access requests and ensuring data compliance. Patients have the right to view and access copies of their own EHR. While a majority of patients trust the NHS with their data and support its use in quality improvement and research, public awareness about data use is generally very low.^28^ Moreover, while many patients are in favour of automatic access to their own records, GPs’ opinions remain divided.^29^

AI scribes may improve patients’ understanding of their own records through simplified accounts. However, such accounts are not simply a transcript of a conversation. They are an *analysis* of a clinician’s diagnostic reasoning; an *interpersonal object*, signalling to the patient what is taken seriously, what is named, and what is left unsaid; and an *interprofessional object* — a way of communicating with colleagues who learn to ‘read’ each other’s shorthand, hedging, and emphasis. AI scribes risk undermining this tripartite by reducing records to a simple object, rather than part of a relational web of knowledge.

This is heightened by the commercial model driving AI tools. Consultation notes have become valuable commercial assets — to the concern of GPs.^26^ AI scribes are also mechanisms for producing data in a form that is attractive to companies, commissioners, and regulators. Commercial tools privilege data — and therefore completeness and length — over interpretability and collegial readability, thus reducing professional utility.

Detailed, verbatim records also pose concerns about the governance of third-party information. Tangential references to other patients are often made in consultations. Such data, usually shared in confidence, may form an essential part of an individual’s care, but must be redacted when health records are requested or made visible online to protect confidentiality. As such, much like with the rollout of patients’ access to their records, AI tools may introduce unintentional safeguarding risks for vulnerable individuals, with additional work required by GPs to mitigate them.

## The ‘system’ argument

Advocates for AI scribes project the efficiency arguments for individual consultations to the whole system, with hopes that small-scale improvements will compound at scale. However, the introduction of such technologies occurs within a digital ecosystem already characterised by multiple platforms, portals, alerts, dashboards, templates, and icons. Each of these products was also introduced with the same belief that they would achieve significant system efficiencies. While some tools have aided administrative and communication tasks, workflow distribution processes, and overall system integration, the predicted benefits have also frequently failed to materialise. The proliferation of technologies has contributed to a hugely pressured environment. Integrating and using new digital technologies demands great cognitive, emotional, or organisational effort. When this becomes overwhelming for staff, they suffer ‘technostress’ and a sense of diminished wellbeing.^5,29^ AI scribes risk exacerbating this pressure by diverting attention from relational interactions to ensuring the system has not misunderstood, omitted, or over-recorded information.^19^

Moreover, the erosion of professional ownership and meaning introduced through digital systems can generate emotional and professional strain if tools are poorly aligned with how clinicians conceptualise good practice. Clinicians who value relational practice may find this aligns poorly with the patterns of care encouraged by AI models, resulting in reduced professional satisfaction. In an already crowded digital environment AI scribes are at risk of functioning less as a liberating innovation and more as another layer of technological demand that exacerbates fatigue and inequities, undermines autonomy, and subtly reshapes what it means to do clinical work. We provide a fictional illustrative vignette in Box 1, based on the experiences of clinical authors.

Box 1. **Illustrative vignette**

My afternoon clinic is running late as usual. The next patient, Sara, has booked about ‘ongoing pelvic pain’. I’ve met Sara several times but can’t remember seeing her for pain. Her record includes codes for ‘domestic violence’ and ‘type 2 diabetes’. I remember she’s a refugee and attends with an advocate and interpreter, usually her sister, who is also registered at our practice.

She’s been seen twice in the last month for abdomino-pelvic pain. My colleagues have captured the encounters with an AI scribe. There’s a page of dense text before me. I’m behind already so just call her in. I’ll have to make my own assessment.

Sara enters with her sister. I ask if she is happy with me using an AI scribe. She nods. I start it. Her sister interjects — she’s bringing her 15-year-old daughter to see me next week about her acne, but her mum is worried she’s self-harming. Could I please talk to her about it — oh and don’t mention she’s said anything.

Sara describes her pain. I suggest it sounds like possible endometriosis or fibroids. I ask about pain during sex. She’s distressed and embarrassed. I ask to examine her, but she declines. She tells me my colleagues have done this — why am I not aware? She asks what happened about the tests they said they’d arrange. I can’t find this in the dense page of text, so frustratingly explain we may have to do them again. I suggest a scan, blood tests, and the contraceptive pill. Sara declines the last but agrees to trial tranexamic acid. She is tearful and I pass her a tissue, holding the silence as she thinks. I suggest we meet again to see if the tranexamic acid has helped. She nods.

As they leave, her sister asks where the AI notes will go — will anyone see them?

I review the notes. I see the information about the sister and niece is recorded on Sara’s record; ‘endometriosis’ and ‘fibroids’ have joined Sara’s list of coded problems; the consultation label is ‘abdominal pain’ with no mention of pelvic pain or menstrual symptoms; and there is a long page of dense text, which does not sound like either of us. There is no mention of the distress and embarrassment caused by intimate questions; no reference to the look of shock and discomfort evoked by my suggestion of the contraceptive pill, nor the multiple, eloquent glances exchanged between Sara and her sister or their Urdu interactions. The encounter is superficially (and incorrectly) descriptive. The dialogue about the previous test requests is prominent, as is the patient’s concern about why I don’t already know the relevant information.

There is no humanity, no cultural sensitivity, and no representation of my relational knowledge of her family. Most importantly, little indicates the emotion and uncertainty of the complex interaction.

I sigh and begin to rewrite — remembering belatedly to change the coded problems from the summary.

## Conclusion

While we acknowledge the potential for AI scribes in general practice, we temper this with the practical, layered, and value-laden considerations described above. First, efficiency savings in information recording are countered by the failed efficiencies built into in an ever-increasing transition towards transactional, distributed, and fragmented care. Second, the data-sharing benefits of enhanced classification and codification are questionable in the context of general practice, where the emphasis is often on symptomatic presentation, situational circumstance, or biomedical diagnosis. Third, we are concerned about the risks posed by data sharing and governance, which present real dangers to patients. Last, we worry that commercial interests will drive diminished relational care and professional values, imparting pressures on the wellbeing of individual practitioners and teams. We would appeal to developers and innovators to work *with* practitioners and researchers to ensure such technologies are co-designed, produced, and implemented with appropriate governance around safe and effective application. We hope this would enable a positive future for such technologies within general practice encounters.
